# Variability in urinary phthalates, phenols, and parabens across childhood and relation to adolescent breast composition in Chilean girls

**DOI:** 10.1016/j.envint.2022.107586

**Published:** 2022-10-19

**Authors:** Lara S. Yoon, Alexandra M. Binder, Ana Pereira, Antonia M. Calafat, John Shepherd, Camila Corvalán, Karin B. Michels

**Affiliations:** a Department of Epidemiology, Fielding School of Public Health, University of California, Los Angeles, 650 Charles E. Young Drive South, Los Angeles, CA 90025, USA; b Cancer Epidemiology Program, University of Hawaii Cancer Center, 701 Ilalo Street, Honolulu, HI 96813, USA; c Institute of Nutrition and Food Technology, University of Chile, Macul, Santiago 7830490, Chile; d Division of Laboratory Sciences, National Center for Environmental Health, Centers for Disease Control and Prevention, 4770 Buford Highway, Mailstop F17, Atlanta, GA 30341, USA; e Population Sciences in the Pacific Program, University of Hawaii Cancer Center, 701 Ilalo Street, Honolulu, HI 96813, USA; f Institute for Prevention and Cancer Epidemiology, Faculty of Medicine and Medical Center, University of Freiburg, Elsässerstraße 2, 79110 Freiburg, Germany

**Keywords:** Endocrine disruptors, Breast density, Puberty, Windows of susceptibility

## Abstract

**Background:**

Epidemiologic evidence suggests that environmental factors acting as endocrine disrupting chemicals (EDCs) are associated with mammographic breast density and the risk of breast cancer. Exposure to EDCs during puberty, a period of rapid breast development, may affect susceptibility to breast carcinogenesis.

**Methods:**

In a cohort of 366 Chilean adolescents from the Growth and Obesity Cohort Study, we evaluated the relation between urinary concentrations of 15 suspected EDC biomarkers across three pubertal time points (Tanner breast stage 1 (B1), 4 (B4), and 1-year post-menarche) and breast fibroglandular volume (FGV; percent FGV [%FGV] and absolute FGV [aFGV]) and total breast volume (tBV) at 2-years post-menarche. We used linear mixed models to test differences in creatinine-corrected EDC biomarker concentrations at B4 and 1-year post-menarche compared to B1 and calculated intraclass correlation coefficients (ICC) of EDC concentrations across time points to appraise the consistency of measurements. We fit multivariable generalized estimating equations (GEEs) to evaluate windows of susceptibility for the association between log_10_-transformed EDCs and log_10_-transformed breast outcomes. GEEs were adjusted for age, body fat percentage, total caloric intake, and maternal education.

**Results:**

Urinary EDC biomarker concentrations highly varied across pubertal time points (ICC range 0.01–0.30). For 12 EDCs, biomarker concentrations decreased over time. Triclosan measured at 1-year post-menarche was inversely associated with %FGV at 2-years post-menarche (β = −0.025, 95 % confidence interval = −0.041, −0.008). Mono(2-ethyl-5-carboxypentyl) phthalate and the sum of di(2-ethylhexyl) phthalate metabolite concentrations at B4 were positively associated with aFGV and tBV at 2-years post-menarche. No measured phenols were associated with aFGV and tBV, while no measured parabens were associated with %FGV and aFGV.

**Conclusions:**

Our study suggests relatively high variability in EDC biomarker concentrations across the peripubertal time period. We also found evidence to suggest that there may be pubertal windows of susceptibility to select EDCs for the association with adolescent breast density.

## 1. Introduction

Endocrine disrupting chemicals (EDCs) are exogenous agents that interfere with processes in the endocrine system, including synthesis and metabolism of hormones (Diamanti-Kandarakis et al., 2009). Recent trends in breast cancer risk suggest that exposure to putative EDCs, including phthalates, parabens, and phenols, may play a role in increasing incidence of breast cancer (Wan et al., 2021). The question of how EDCs are associated with breast cancer has been evaluated in a limited number of animal and epidemiologic studies (Gore et al., 2015). In animal models, exposure to bisphenol A (BPA) has resulted in increased mammary gland growth, greater cell proliferation, and more tumor multiplicity (Lamartiniere et al., 2011; Markey et al., 2001; Vandenberg et al., 2008). Prenatal exposure to butyl benzyl phthalate in rats was associated with increased mammary gland susceptibility to carcinogens through modulation of gene expression (Moral et al., 2011). Epidemiologic evidence for an association between EDCs and breast cancer risk is inconsistent. Medium-sized (N < 500) studies of breast cancer cases and controls in adult populations of Mexican and Native Alaskan women have found positive associations between certain phthalate biomarkers, including monoethyl phthalate (MEP) and mono (2-ethylhexyl) phthalate (MEHP), and breast cancer risk (Holmes et al., 2014; López-Carillo et al., 2010). In a larger nested-case control study within the Women’s Health Initiative, urinary concentrations of 13 phthalate biomarkers were not associated with breast cancer risk (Reeves et al., 2019). Recently, a nested case-control study within the Multiethnic Cohort Study reported suggestive associations for increased breast cancer risk among women with a higher ratio of MEHP to oxidative di(2-ethylhexyl) phthalate (DEHP) metabolites compared to those with a lower ratio (Wu et al., 2021). Oxidative DEHP metabolites were presumed to have a lowerF internal body burden than MEHP, the hydrolytic DEHP metabolite, and thus would have lesser physiological effect on breast carcinogenesis (Wu et al., 2021). In a US population-based study with 18 years of follow-up, higher urinary concentrations of methylparaben and propylparaben were associated with a 30 – 50 % increase in breast cancer risk; the association was stronger among women with body mass index (BMI) < 25.0 kg/m^2^ (Parada et al., 2019). Studies of Polish and American women have not found evidence to link urinary bisphenol-A concentrations to breast cancer risk (Aschengrau et al., 1998; Trabert et al., 2014).

Endocrine disruptors, ubiquitous in personal care products, industrial materials, food packaging, and pharmaceuticals (Birnbaum et al., 2012; Guo and Kannan, 2013; Kelley et al., 2012), have the potential to interfere with breast carcinogenesis through their action on hormone receptors (Gore et al., 2015). EDCs have been implicated in modulation of estrogenic and anti-estrogenic activity, modification of development of mammary tissue, and inhibition of sex steroids metabolism (Gore et al., 2015). A strong risk factor for breast cancer is high mammographic breast density, a measure of the amount of fibroglandular tissue in the breast (Boyd et al., 2010). Breast tissue development begins early in the lifecourse and may be particularly sensitive to environmental perturbation during puberty, a period of rapid growth and cellular differentiation driven by hormones and other growth factors (Russo, 2016). Exposure to EDCs during this window of sensitivity may have outsized impact on breast development and subsequent susceptibility to breast cancer, as pubertal breast development is thought to be an important determinant of adult mammographic density (Ghadge et al., 2021). While the effect of exposure to EDCs on breast density is understudied, several studies of adolescents have found that EDCs are positively related to age at pubertal onset, including pubarche and menarche, which in turn are related to breast cancer risk (Bodicoat et al., 2014; Houghton et al., 2019; Wolff et al., 2010).

We have previously found associations between childhood exposure to select phenols and phthalates and breast composition at Tanner breast stage 4 (B4), a late pubertal stage, in a cohort of Chilean adolescents. Specifically, B4 breast density was higher among girls with higher levels of monocarboxy-isooctyl phthalate (MCOP) measured at Tanner breast stage 1 (B1), a pre-pubertal stage, and cross-sectionally at B4, and positively associated with MEP concentrations measured cross-sectionally at B4 (Binder et al., 2018). In this study, we expanded on our earlier analysis by increasing the sample from 200 to 366 participants, increasing the number of time points at which the EDC biomarkers were assessed from two to three, and utilizing a post-menarche measurement of breast density, when the breast has reached maturity. Breast density is thought to peak in young women following menarche; high density at this age may define breast density trajectories throughout the life course (McCormack et al., 2010). The goals of this study were to assess the variability of EDC biomarker concentrations across three peripubertal time points and to evaluate the relation between EDC biomarker concentrations breast composition at 2-years post-menarche. This study provides additional insight into potential windows of susceptibility to EDC exposures during childhood on adolescent breast density.

## 2. Materials and methods

### 2.1. Study population

Participants in this study were part of the Growth and Obesity Cohort Study (GOCS), an ongoing longitudinal cohort study of children in Santiago, Chile. GOCS began in 2006 and included 1,196 children aged 2.5–4 years enrolled in preschool at the National Board of Preschool Council Program (Junta Nacional de Jardines Infantiles) (Corvalán et al., 2009). Participants in the study met the following criteria: (1) singleton birth born at term (37–42 weeks), (2) birthweight of ≥ 2500 and < 4500 g, and (3) without health conditions that might affect growth (e.g., metabolic or endocrine disorders). Of the children initially enrolled in the cohort, approximately half (n = 601) were female. Once enrolled, participants visited the Instituto de Nutrición y Tecnología de los Alimentos (INTA) Health Clinic at the Universidad de Chile in Santiago, Chile at least one per year; in 2011, the visit frequency increased to twice per year to better capture pubertal maturation. At the clinic, trained dietitians evaluated anthropometry, bioimpedance, and pubertal development (thelarche, menarche, Tanner staging) in the children. Biological specimens were collected at defined follow-up time points corresponding to pubertal breast development: Tanner breast stage B1 (pre-pubertal), Tanner breast stage B4 (late puberty), and 1-year post-menarche. Limited socioeconomic, demographic, and behavioral information was also collected via questionnaires completed by the mothers of the children. Diet was assessed using the in-person 24-hour interview recall method every-six months by trained dietitians beginning in 2013 (Gaskins et al., 2017). Breast density was measured using dual X-ray absorptiometry at 2-years post-menarche among 525 girls. The current study includes 366 girls with a breast assessment at 2-years post-menarche and at least one fasting urine sample collected at B1, B4, or 1-year post-menarche (Supplemental Figure 1). The study protocol was approved by the University of Chile Ethics Committee at INTA and the University of California, Los Angeles Institutional Review Board. Written informed consent was obtained from all parents or guardians of the children at enrollment and again before breast assessment. The analysis of blinded specimens by the CDC laboratory was determined not to constitute engagement in human subjects’ research.

### 2.2. EDC biomarker assessment

Fasting spot urine samples were collected during visits to the clinic at INTA corresponding to three study time points: B1, B4, and 1-year post-menarche. During morning visits to the clinic, each participant collected at least 2 mL of urine in polypropylene sterile containers. The urine was temporarily stored at 4 °C before processing for homogenization of the sample and measurement of urinary density, followed by aliquoting and storage at −80 °C before being shipped to a laboratory for biomarker quantification. This protocol has shown to be effective with regards to temporal stability of metabolites in the urine (Samandar et al., 2009).

We selected 18 suspected EDC urinary biomarkers a priori, including triclocarban, 5 phenols (benzophenone-3, BPA, bisphenol F [BPF], bisphenol S [BPS], triclosan), 4 parabens (ethylparaben, butylparaben, methylparaben, propylparaben), and 8 phthalate metabolites (mono(2-ethyl-5-carboxypentyl) phthalate [MECPP], mono(2-ethyl-5-hydroxyhexyl) phthalate [MEHHP], MEHP, mono(2-ethyl-5-oxohexyl) phthalate [MEOHP], MEP, mono-isobutyl phthalate [MiBP], mono-*n*-butyl phthalate [MBP], and the nonspecific metabolite of several phthalates mono-3-carboxypropyl phthalate [MCPP]). Urine samples collected at B1 and B4 from 200 randomly selected girls (400 samples total) were processed at the CDC National Center for Environmental Health Laboratory using on-line solid phase extraction-high performance liquid chromatography-isotope dilution-tandem mass spectrometry as previously described (Silva et al., 2007; Ye et al., 2005). Quality control pooled human urine materials were analyzed along with standards, blanks, and study samples. The limits of detection (LOD) ranged from 0.1 to 1.7 ng/mL depending on the analyte (Silva et al., 2007; Ye et al., 2005). Additional funding supported the analysis of the remaining urine samples from 166 girls collected at B1 (93 samples), B4 (133 samples), and 1-year post-menarche (232 samples) at the Mount Sinai CHEAR Network Laboratory Hub using a previously described protocol (Mazzella et al., 2021). EDC biomarkers needed to have been measured by both the CDC laboratory and the Mount Sinai laboratory in order to be included in this analysis. One EDC biomarker, monobenzyl phthalate (MBzP), was excluded from this analysis and results will be presented elsewhere. Creatinine quantification for all samples was performed at Mount Sinai. A subset of 40 samples collected at B1 and B4 and initially analyzed at the CDC laboratory was also analyzed at the Mount Sinai laboratory to evaluate agreement between laboratories. Then, we calculated the intraclass correlation coefficient (ICC) using a one-way random effects model measuring absolute agreement with multiple raters/measurements (McGraw and Wong, 1996; Shrout and Fleiss, 1979). A total of 15 EDC biomarkers were used in the analyses; the mean ICC for biomarker pairs was 0.87. Three EDC biomarkers with ICC < 0.75 and with more than 50 % replicates below the laboratory-specific LOD for both samples were excluded from further analysis (BPF, butylparaben, triclocarban). EDC biomarker concentrations below the laboratory-specific LOD were imputed a value of the LOD/sqrt(2) (Hornung and Reed, 1990).

Prior to analysis, we standardized the distribution of EDC biomarker concentrations across assay batches. The samples analyzed by both labs were used to estimate the difference in the mean and relative standard deviation (SD) in biomarker concentrations between the two labs. These estimates were then used to shift the mean and scale the SD among the full sample group analyzed at CDC to that of the samples analyzed at Mount Sinai, assuming the true distribution of concentrations between the two labs was the same and there were no differences in participant characteristics for the samples analyzed at different labs. We also calculated the molar summation of several biomarkers: DEHP metabolites (MEHP, MEHHP, MECPP, MEOHP), ∑high- molecular weight phthalates (high-MWP) (MCPP, MECPP, MEHHP, MEHP, MEOHP), ∑low-MWP (MEP, MiBP, MBP), ∑phenols (benzophenone-3, BPA, BPS, triclosan), and ∑parabens (ethylparaben, methylparaben, propylparaben) by dividing each biomarker concentration by its molar mass and then summing the individual concentrations.

### 2.3. Breast composition assessment

Breast assessments were completed when the girls were 2-years post-menarche. Dual energy X-ray absorptiometry (DXA) was used to quantify dense breast tissue volume (fibroglandular volume; FGV) based on a breast scanning protocol developed by Shepherd and colleagues the University of California, San Francisco (Shepherd et al., 2006). In this method, each breast was scanned with the Prodigy DXA system software (version 13.6, series 200674; GE Healthcare). The DXA system was continuously calibrated throughout the study using a quality control breast phantom. Values from the left and right breast were averaged to obtain single measures of absolute FGV (aFGV; cm^3^) and total breast volume (tBV; cm^3^). We derived percent FGV (%FGV; %) by calculating the proportion of absolute FGV among total breast volume. The DXA protocol has high validity and precision for breast density assessments among adolescent girls and is frequently used to evaluate bone density in children (Crabtree et al., 2014; Shepherd et al., 2008). The high validity of these breast outcomes has been previously reported (Pereira et al., 2017). All breast composition assessments were log_10_-transformed prior to analyses.

### 2.4. Covariates

Demographic, anthropometric, and nutritional information was collected during follow-up visits to the INTA health clinic. Body fat percentage was measured using bioelectrical impedance measurements (Tanita-BC-418 MA, Tanita-Corporation, Tokyo, Japan) and presented as a continuous measure. Body fat percentage was also categorized (underfat/normal, overfat, obese) based on Tanita age- and sex-specific body fat reference curves (Cediel et al., 2016; McCarthy et al., 2006). Age at menarche was determined via phone interviews completed by study dietitians every 3 months during puberty and confirmed at the subsequent study visit. Information on birth mode (vaginal, caesarean), duration of predominant breast-feeding (<3 months, 3–6 months, >6 months), and maternal education (secondary education or less, more than secondary education) was collected via interviews with the mothers of the participants. Total caloric intake (g/day) was measured using 24-hour dietary recalls at each clinic visit and averaged across the recalls that occurred prior to each study time point (B1, B4, 1-year post-menarche) for each girl to reduce random measurement error (Willett, 2012). Missing covariate data were imputed using last observation carried forward if available followed by mean or median imputation.

### 2.5. Statistical analysis

To assess temporal variability in EDC biomarker concentration, we calculated geometric means and 95 % confidence intervals (CI) at each study time point. The geometric mean is less influenced by extreme outliers than other measures of central tendency (Rosner, 2016). ICCs and corresponding 95 % CI were calculated from a one-way random effects model for consistency to compare variability of each log_10_-transformed EDC biomarker concentration across the three study time points (B1, B4, 1-year post-menarche) (Shrout and Fleiss, 1979). The ICC ranges from 0 to 1; a higher ICC indicates less intra-individual variability (Rosner, 2016). Individual linear mixed models (LMM) with random intercepts were used to evaluate differences in log_10_-transformed EDC biomarker concentrations at B4 and 1-year post-menarche compared to B1. These LMMs allow for intra-individual correlation across timepoints. Spearman correlation coefficients were calculated between log-transformed EDC biomarkers separately for each time point. EDC concentrations were creatinine-corrected to account for dilution and are presented in units of μg/g creatinine or μmol/g creatinine. For analyses of temporal variability, we corrected for creatinine using the classical adjustment method by dividing urinary EDC biomarker concentration by creatinine concentration (O’Brien et al., 2017).

To evaluate windows of susceptibility for the association between log_10_(ng/ml)- EDC biomarker concentrations and breast outcomes, we fit generalized estimating equation (GEE) models with an identity link and independent correlation structure. This GEE approach is based on the multiple informants method and models subject-specific patterns of EDC biomarker concentrations (repeated measures) in relation to the breast composition outcome (Chen et al., 2015; Sánchez et al., 2011). The multiple informants GEE approach can be used to examine whether the exposure of interest is associated with the outcome in the same manner for each study time point and provides a single coefficient estimate for each time point (Sánchez et al., 2011). We examined the significance of the interaction between study time point and log_10_-EDC biomarker concentration on breast outcomes (F test). All models were adjusted for time-varying (age (years; continuous), body fat percent (continuous), total caloric intake (kilocalories (kCal) per day; continuous)) and fixed (maternal education (categorical)) factors selected a priori as potential confounders using directed acyclic graphs (Tennant et al., 2021). We used non-creatinine-adjusted EDC biomarker concentrations in the multivariable GEE analyses and additionally included creatinine as covariate in the models (Barr et al., 2005).Therefore, beta coefficients represent the association between log_10_(ng/ml)- EDC concentrations and log_10_-transformed breast outcomes. Because exposure often occurs to mixtures and not isolated EDCs, many EDC biomarkers are thus likely to be correlated. We therefore did not include more than one EDC biomarker in a model to avoid inducing large variance. We did not adjust for multiple comparisons for coefficients and confidence intervals within the GEE models due to high shared variation between EDCs and to avoid reducing power (Rothman, 1990). However, we did adjust p-values for analyses evaluating the significance of the interaction between study time point and log_10_-EDC biomarker concentration using the Benjamini-Hochberg procedure; false discovery rate (FDR) < 0.05 was considered significant (Benjamini and Hochberg, 1995). All analyses were performed in R version 4.0.4 (R. Core Team, 2013).

## 3. Results

### 3.1. Study population characteristics

Participant characteristics at each study time point are presented in Table 1. Among the 366 girls with breast assessments at 2-years post-menarche included in the study, 293 provided a urine sample at B1; 333, B4; and 232, 1-year post-menarche. A total of 185 girls provided a urine sample at all three time points. Girls included in the study did not noticeably differ from the 159 girls excluded from the study for missing breast composition assessments with respect to key study characteristics (Supplemental Table 1). Of the participants included at each of the study time points, the proportion providing a urine sample on at least one other time point was moderate to high. For instance, among girls who provided a urine sample at B4, 65.8 % also provided one at 1-year post-menarche; among the girls with a urine sample at 1-year post-menarche, 84.9 % had provided one at B1. Participants were, on average, 7.9 years old (SD 0.45) at B1, 11.4 years old (SD 0.9) at B4, and 13.4 years old (SD 0.8) at 1-year post-menarche. Overall, participants’ mean body fat percentage increased from B1 to 1-year post-menarche (25.6 % to 30.6 %), with the greatest proportion of obese girls at 1-year post-menarche. A majority of mothers of the participants reported secondary education or less, 3–6 months of predominant breast feeding, and vaginal births. Average caloric intake of the girls ranged from 1,745 to 1,873 kCal/day across the study time points.

### 3.2. Temporal variability of EDC biomarkers

Creatinine-adjusted EDC biomarker geometric means and standard deviations by study time point are presented in Table 2. Apart from BPA, the distribution of the log_10_-transformed and creatinine-adjusted EDC biomarker concentrations at B1, B4, and 1-year post-menarche did not significantly differ between GOCS girls included in the analysis and girls excluded from the analysis (Supplemental Figure 2). Overall, results from linear mixed models suggest that EDC concentrations were significantly lower at B4 and 1-year post-menarche compared to B1 for individual EDCs (methylparaben, ethylparaben, propylparaben, and all phthalate metabolites) and for summed EDC groupings (∑Phenols, ∑Parabens, ∑Low-MWP, and ∑High-MWP) using a 5 % level of statistical significance However, benzophenone-3 concentrations were higher at B4 and 1-year post-menarche compared to B1. Spearman correlations of individual EDC biomarker concentrations within study time points ranged from low (close to 0) to high (close to 1) depending on the EDC biomarker grouping (Supplemental Figure 3, Supplemental Figure 4, Supplemental Figure 5). In general, EDC biomarkers within the same chemical class were more likely to be highly correlated with each other than with biomarkers from other chemical classes. For instance, DEHP metabolites highly correlated with each other; parabens highly correlated with other parabens. This pattern was consistent within each study time point. We also found evidence to suggest high variability of EDC biomarker concentrations across study time points (Table 2). The ICC for phenols ranged from 0.01 to 0.11; for parabens, 0.07 to 0.15; for phthalates, 0.06 to 0.30. Additional graphical representation of EDC concentration over time is provided in Supplemental Figure 6.

### 3.3. Association of EDC concentrations with breast composition measures

A log_10_(ng/ml) increase in benzophenone-3 concentration at B1 was associated with a modest decrease (β:−0.024, 95 % CI: 0.05, 0.000) in log_10_(%FGV) at 2-years post-menarche after adjusting for creatinine, maternal education, age, body fat percentage, and average daily caloric intake (Table 3). However, this trend was not statistically significant. We found an statistically significant inverse association between triclosan at 1-year post-menarche and %FGV at 2-years post-menarche (β:−0.025, 95 % CI: −0.41, −0.008). The interaction between triclosan biomarker concentration and study time point was statistically significant, suggesting differences in associations between triclosan concentrations and %FGV across B1, B4, and 1-year post-menarche (*p* = 0.007). However, adjustment for multiple comparisons suggested no such significant difference (FDR = 0.12). No other EDC biomarkers measured at B1 or 1-year post-menarche were associated with differences in %FGV at 2-years post-menarche, nor were any EDC biomarkers measured at B4. We also did not report other statistically significant interactions between EDC biomarker and study time point on %FGV at 2-years post-menarche.

A higher concentration of MBP measured at B1 was associated with statistically significant lower aFGV at 2-years post-menarche (β: −0.09, 95 % CI: −0.15, −0.024) (Table 4). Higher aFGV at 2-years post menarche was also statistically associated with higher concentrations of MECPP (β: 0.055, 95 % CI: 0.006, 0.104) and ∑DEHP metabolite biomarkers (β: 0.053, 95 % CI: 0.003, 0.104) measured at B4. The association between EDC biomarker concentration and aFGV at 2-years post-menarche was significantly modified by study time point for several phthalates (MBP, MEHHP, and ∑DEHP) in models adjusted for potential confounders (p < 0.05). After adjustment for multiple comparisons, only MBP remained statistically significant (FDR = 0.047) (Table 4).

Biomarker concentrations of phenols measured at any time point were not associated with tBV (Table 5). Propylparaben measured at 1-year post menarche was associated with modestly higher tBV at 2-years post menarche (β: 0.021, 95 % CI: 0.001, 0.041), and the relation of propylparaben to tBV was significantly modified by study time point (p = 0.038). Several phthalate biomarkers MBP, MECPP, MEHP) were linked to differences in tBV; study time point significantly modified the associations (p < 0.05). However, none of the summed phthalate groupings were significantly associated with tBV. Similarly to %FGV (Table 3) and aFGV (Table 4), no chemicals remained significantly associated with TBV after adjustment for multiple comparisons.

## 4. Discussion

In this longitudinal study of adolescent Chilean girls, urinary concentrations of select EDC biomarkers measured across different stages of puberty were weakly and inconsistently associated with breast outcomes measured at 2-years post-menarche. These associations were not consistent across time: for EDC biomarkers significantly associated with breast outcomes, those measured at B1 were generally associated with lower breast density and volume, while those measured at B4 and 1-year post-menarche were largely associated with higher breast density and volume. Our results also suggest differences in EDC biomarker concentration across time. For the majority of EDC biomarkers we evaluated, concentrations at B4 and 1-year post-menarche were significantly lower than concentrations at B1. Taken together, these findings suggest that breast development may have windows of susceptibility to EDC exposure throughout puberty. However, given the number of hypotheses tested in our analyses, we cannot rule out the possibility of significant findings by chance.

### 4.1. Variability in EDC biomarker concentration across puberty

We reported geometric mean concentrations of creatinine-adjusted EDC biomarkers across time points corresponding to age 8 (B1), 11.5 (B4) and 13.5 (1-year post-menarche) years. Overall, urinary concentrations of EDC biomarkers were similar in our study population to those observed in other studies of young children or adolescents in the United States (U.S.), China, Sweden, and Mexico (Calafat et al., 2008; Wolff et al., 2010; Buttke et al., 2012; Lewis et al., 2013; Wang et al., 2014; Larsson et al., 2014; Stacy et al., 2016). These observed differences in EDC biomarker concentration may reflect true geographic differences in population exposure to certain EDCs. For example, we observed slightly lower concentration of BPA among the girls in our study (1.4 – 1.6 μg/g creatinine) compared to studies of U.S. children of similar age (1.1 – 4.2 μg/g creatinine), which might suggest decreased exposures through sources such as ultra-processed food (UPF) packaging (Buttke et al., 2012; Stacy et al., 2016). EDCs such as BPA, DEHP, and other phthalates (e.g., benzylbutyl phthalate) can be present in UPF packaging such as plastic containers and food lining (Pacyga et al., 2019). UPFs account for roughly a quarter of total energy intake (kCal) in the general population in Chile (Cediel et al., 2021). In comparison, data from the National Health and Nutrition Examination Survey suggests that more than 50 % of total energy intake among the U.S. general population and more than 65 % of total energy intake among children and adolescents comes from UPF (Baraldi et al., 2018). We also observed higher concentrations of paraben biomarkers (ethylparaben, methylparaben) in the Chilean girls compared to Swedish and Danish children of similar age, which might relate to differences in public and regulatory focus on parabens in the European Union compared to Chile (Frederiksen et al., 2013; Larsson et al., 2014).

In the present study we observed decreasing EDC biomarker concentrations over time. With the exception of benzophenone-3 and BPA, creatinine-corrected concentrations of individual phenols, parabens, and phthalate biomarkers were lower at B4 and 1-year post-menarche compared to B1. This trend is somewhat unexpected, as we might anticipate higher concentrations of certain parabens and phthalates biomarkers at older ages with increasing use of cosmetics and other personal care products. However, creatinine also increases with age; therefore, creatinine-adjusted concentrations of EDC biomarkers may decline with increasing age (Barr et al., 2005). In contrast to our analysis, prior studies of Danish children reported higher concentrations of MEP among girls at older ages and more advanced pubertal stages (B4 and B5) compared to younger girls and those at less advanced stages (e. g., B1) (Frederiksen et al., 2012, 2011). Notably, creatinine was either not measured or not mentioned in these studies. A U.S. study observed higher metabolite concentrations of certain high-MWPs (MCOP, MCNP) among children aged 6–11 years compared to adolescents (12–19 years); these concentrations were also corrected for creatinine (Calafat et al., 2011). Though we did not quantify MCOP and MCNP in this analysis because they were only measured at one lab, we did observe a similar trend of higher concentrations at earlier pubertal stages (i.e., younger ages) among metabolites of other high-MWP (MCPP, MECPP, MEHHP, MEHP, MEOHP). While these studies confirm exposure to EDCs in childhood, it is difficult to disentangle changes in exposure to these chemicals from physiological differences such as changing body size.

Results from our study suggest relatively high variability in biomarker concentrations across the study period for all EDC biomarkers. Our ICCs generally agree with those reported in cohort studies in populations of children, with most studies reporting low ICCs, particularly when the period of time between urine collection is greater than several months. The HOME study of U.S.-based pre-school aged children reported relatively high variability of BPA and phthalate metabolite concentrations (ICC range 0.09–0.39) over an 8-year study period, with MEP exhibiting the lowest variability across time (Stacy et al., 2016; Teitelbaum et al., 2008; Watkins et al., 2014). Overall, other studies which have evaluated longitudinal EDC concentration in childhood or adolescence find relatively high variability across time, particularly when the period between sample collections is greater than several months (Johns et al., 2015; Kim et al., 2021; Stacy et al., 2016). We also observed less temporal variability and lower concentrations of urinary MEHP, the hydrolytic metabolite of DEHP, across time compared to the other three DEHP metabolites (MECPP, MEHHP, MEOHP) measured in the study. Other observational studies have reported similar relative variability (ICC range 0.30–0.50) and lower concentration of MEHP relative to the oxidative DEHP metabolites ((Adibi et al., 2008); Becker et al., 2004; Cantonwine et al., 2014; Chen et al., 2017; Fisher et al., 2015; Navaranjan et al., 2020). The reasons for the lower variability of MEHP compared to other DEHP metabolites are unclear as MEHP has a shorter half-life compared to oxidative DEHP metabolites (Kavlock et al., 2002). It is possible that DEHP exposure is relatively stable across childhood and the distribution of oxidative DEHP metabolites concentrations relates to changes in the body’s metabolic activity with age and body size (Becker et al., 2004; Silva et al., 2006).

### 4.2. Association of EDC biomarkers with breast outcomes

In our study, select phenols and phthalates biomarkers, but not parabens, measured across puberty demonstrated weak but significant associations with breast outcomes at 2-years post-menarche. Our results for the associations between these EDCs measured at B1, B4, and 1-year post-menarche and breast outcomes at 2-years post-menarche are not consistent with our prior study, which measured breast outcomes at B4. We had previously found an inverse association between triclosan concentration at B1 and B4 and aFGV at B4, a positive association between MEP concentration at B4 and aFGV at B4, and a non-linear association for the relation of BPA to aFGV and MCNP to tBV (Binder et al., 2018). There are several major differences in this study and our prior study. In the current study, our sample size increased by more than 150 girls, increasing our study power. Girls included in this study differed from those in our prior study with respect to BMI Z-score: median BMI Z-score at B1 and B4 were 0.85 and 0.88, respectively. In contrast, girls included in the prior study had median BMI Z-score of −0.1 at B1 and 0.1 at B4. It is likely that the higher BMI Z-score in the current study sample better reflects the nutritional intake and obesity status of the cohort and of Chilean girls overall. While both studies adjusted for body size, we were able to further adjust for total caloric intake to account for any residual confounding in the current study. We also evaluated three time points across the peripubertal window representing different periods of development, rather than two. Our breast composition outcome was assessed at 2-years post-menarche, after all the exposure windows. In contrast, our prior study evaluated a simultaneous EDC measure and breast outcome assessment (B4). Looking at prospective exposure to EDCs such as diethyl phthalate, the parent compound of MEP, and later breast outcomes may have allowed us to better characterize the temporal nature of any potential effect of EDCs on breast development. Finally, we were able to capture a measure of breast density composition post-puberty when the breast has reached maturity (McCormack et al., 2010). Unpublished analyses from our cohort suggest a correlation between breast density measures at B4 and a 2-years post-menarche: girls in the highest category of breast density at B4 are likely to remain in the highest category at 2-years post-menarche. However, aFGV and tBV overall are significantly higher in girls at the 2-year post-menarche timepoint compared to the B4 timepoint.

While to our knowledge no other cohorts have evaluated the EDC-breast composition link in adolescents, several other studies have evaluated the relation between these chemical biomarkers and the timing of breast development. Evidence from longitudinal studies in the USA and UK suggests that timing of breast development is related to breast composition: earlier thelarche (i.e., first breast development) and greater time between thelarche and menarche have been associated with higher adult percent breast density (Houghton et al., 2019; Schoemaker et al., 2017). Moreover, an increase in adult breast density and earlier thelarche are both associated with an increase in breast cancer risk (Bodicoat et al., 2014). It is unknown whether these risk factors are markers for each other or whether they might act through similar mechanisms to influence breast cancer risk. However, we might expect similar associations between EDCs and timing of breast development, and EDCs and breast composition. Support for this theory is inconsistent across other longitudinal cohorts. Two publications from the Breast Cancer and Environment Research Program (BCERP), a multi-ethnic longitudinal cohort study of U.S. girls, report earlier breast development (i.e., age at B2) for higher urinary concentrations of triclosan at age 6–8 years, but later breast development for higher benzophenone-3 and MBzP concentrations at age 6–8 years (Wolff et al., 2014, 2015). The BCERP studies and others have also observed null associations between select EDCs, including low-molecular weight phthalate biomarkers and phenols, and the timing of breast development. A study of 725 Danish girls did not find significant relations of age at B2 to concentrations of 12 phthalate biomarkers (Frederiksen et al., 2012). In the U.S.-based CHAMACOS longitudinal cohort of Latinos, peripubertal concentrations of benzophenone-3 and triclosan were not associated with age at B2 (Harley et al., 2019). Evaluating the literature on the association between EDCs and adolescent breast development should consider the difference in choice of breast or pubertal outcomes, study populations, sample sizes, and the timing of EDC biomarker measurements across studies.

It is notable that our current study was able to measure EDC biomarker concentrations at three different time points across puberty, compared to a single peripubertal window in other cohorts, allowing for examination of specific susceptible peripubertal periods. We observed significant interaction by study time point for the association between certain DEHP metabolites and breast outcomes, with significant positive associations for B4 concentrations of MECPP, MEHHP, and MEOHP and both aFGV and tBV. It is plausible that the breast is more susceptible to EDC exposure during the B4 stage, in which the breast tissue is continuing to differentiate and proliferate, compared to B1, a pre-pubertal stage in which there is less rapid development, or 1-year post-menarche, when the breast is mature (Javed and Lteif, 2013). However, the significant associations observed with aFGV were among the phthalates biomarkers with higher concentrations. A potential explanation for lack of significance at this stage for other phthalates is that we are limited in power to observe associations with lower concentrations. To our knowledge, no other studies have evaluated adolescent breast composition with multiple pubertal exposure time points. Overall, these findings suggest Tanner breast stage B4 as a potential window of susceptibility to DEHP for aFGV.

Finally, a potential explanation for our findings is that EDCs are not associated with breast density in adolescence. Instead, it is possible that the use of multiple statistical tests related to the number of EDC biomarkers and breast outcomes measured increases the likelihood of falsely positive results by chance (Greenland, 2008). We attempted to control for type 1 errors by adjusting p-values from joint hypothesis testing of interaction using the Benjamini-Hochberg method and a standard false discover rate of 0.25 (Benjamini and Hochberg, 1995). However, EDC biomarker concentrations vary significantly across pubertal time periods; it is conceivable that our concentrations do not truly represent the average EDC concentrations among the cohort and that the timing between EDC biomarker measurement and breast outcome measurement significantly precludes any permanent effect. As previously discussed, other cohort studies have reported null associations between EDC biomarkers and important pubertal breast outcomes.

### 4.3. Strengths and limitations

A limitation of our study is potential exposure misclassification from single spot urine collection at each study time point. While urine is the preferred biospecimen for characterizing concentrations of phthalate metabolites, parabens, and phenols as biomarkers, non-persistent chemicals such as EDCs are metabolized quickly and because exposures are likely episodic in nature biomarker concentrations reflect recent exposure (Calafat et al., 2015; Frederiksen et al., 2007). A single urine sample may not accurately represent an entire pubertal period. However, studies suggest that metabolite concentrations of certain phthalates and phenols have moderate to good correlation over time frames of weeks or months in children (Teitelbaum et al., 2008; Watkins et al., 2014). Because EDC exposures are often linked to consistent behavioral and dietary patterns such as use of personal care products and food choices, use of a single urine sample may reasonably reflect an exposure period. Additionally, though we did adjust for multiple comparisons for select joint hypotheses, it is possible that some our reported associations are due to type I error. Urinary metabolite quantification represents a measure of internal dose that accounts for multiple parent compounds and routes of exposure (Johns et al., 2015). We are unable to disentangle the specific source of the metabolite or parent compound exposure, particularly for nonspecific biomarkers that have more than one parent compound such as MCPP. However, biomarkers can be used to estimate the totality of exposure for a relevant time window and may provide a more accurate assessment of EDC exposure than assessment via lifestyle or dietary questionnaire (Lee and Jacobs, 2015).

Our study has several strengths, including the large sample size and ability to prospectively study the relation between EDC exposure and adolescent breast density. We collected repeated samples of urine for EDC biomarkers assessment throughout puberty, which allowed us to examine the associations of interest at more than one time point and identify potential windows of susceptibility. There was moderate loss to follow-up in this study and only marginally varying sample sizes across time points. GOCS has longitudinal covariate information that allowed us to adjust for potential confounders such as maternal education and anthropometry. It is possible that there is residual confounding from diet due to the use of a single measure of total caloric intake. While we were unable to estimate dietary habits that may reflect source exposure to EDCs such as packaged food, we were able to control for factors such as maternal education as a surrogate for socioeconomic status, which may drive dietary choices.

## 5. Conclusions

We found strong temporal variability in urinary concentrations of phthalate metabolites, phenols, and parabens across pubertal time points. Urinary concentrations of a limited number of phenols and phthalates biomarkers across various pubertal stages were associated with differences in adolescent breast outcomes measured at 2-years post-menarche, which suggests potentially varying windows of susceptibility during puberty. However, we cannot rule out findings of chance.

## Supplementary Material

Appendix A. Supplemental Material

## Figures and Tables

**Table 1:** Characteristics of 366 girls in the Growth and Obesity Cohort Study assessed for breast density at 2-years post menarche.

Characteristic	Study Exposure Time Point		
	Tanner Breast Stage 1 (B1)^a^ (n = 293)	Tanner Breast Stage 4 (B4)^a^ (n = 333)	1-Year Post-Menarche (n = 232)
Urine sample provided			
B1	293 (100.0)	261 (78.4)	197 (84.9)
B4	261 (89.1)	333 (100.0)	219 (94.4)
1Y PM	197 (67.2)	219 (65.8)	232 (100.0)
Age, years (mean (SD))	7.9 (0.5)	11.4 (0.9)	13.4 (0.8)
Age at menarche, years (mean (SD))	12.2 (0.9)	12.0 (0.9)	12.4 (0.8)
BMI Z-score (mean (SD))	0.9 (1.1)	0.9 (1.1)	0.9 (1.1)
Body fat percentage (mean (SD))	25.6 (4.4)	26.9 (5.1)	30.6 (5.6)
Body fat percentage category (count (%))			
Underfat/Normal	155 (54.4)	198 (62.3)	55 (40.1)
Overfat	82 (28.8)	68 (21.4)	39 (28.5)
Obese	48 (16.8)	52 (16.4)	43 (31.4)
Maternal education (%)			
Secondary education or less	236 (80.5)	271 (81.4)	192 (82.8)
Greater than secondary education	57 (19.5)	62 (18.6)	40 (17.2)
Duration of predominant breast feeding (count (%))			
< 3 months	90 (30.7)	107 (32.1)	74 (31.9)
3–6 months	171 (58.4)	188 (56.5)	134 (57.8)
>6 months	32 (10.9)	38 (11.4)	24 (10.3)
Birth mode (%)			
Caesarean	85 (29.0)	91 (27.3)	73 (31.5)
Vaginal	208 (71.0)	242 (72.7)	159 (68.5)
Average caloric intake, kCal (mean (SD))	1878.8 (490.7)	1873.0 (457.6)	1745.1 (446.4)

a Tanner stages correspond to pubertal breast development stages B1 (pre-pubertal) and B4 (late puberty).

**Table 2:** Creatinine-adjusted urinary phenol, paraben, and phthalate biomarker geometric means (95% confidence interval) by study time point.

N	Tanner Stage B1^a^ 293	Tanner Stage B4^a^ 333	1-Year Post-Menarche 232	ICC (95 % CI)
Phenols				
Benzophenone-3	31.3 (27.7, 35.4)	50.5 (43.6, 58.5)^b^	47.0 (40.0, 55.4)^b^	0.1 (0.0, 0.2)
BPA	1.6 (1.4, 1.7)	1.4 (1.3, 1.6)	1.6 (1.4, 1.7)	0.1 (0.0, 0.2)
BPS	0.5 (0.5, 0.6)	0.5 (0.4, 0.6)	0.4 (0.3, 0.4)^b^	0.0 (0.0, 0.1)
Triclosan	12.3 (10.5, 14.5)	11.2 (9.6, 13.1)	10.1 (8.2, 12.5)	0.1 (0.0, 0.2)
∑Phenols^c^	0.2 (0.2, 0.3)	0.3 (0.3, 0.4)^b^	0.3 (0.3, 0.4)^b^	0.1 (0, 0.2)
Parabens				
Ethylparaben	1.8 (1.5, 2.2)	1.1 (1, 1.4)^b^	1.0 (0.8, 1.3)^b^	0.1 (0.0, 0.2)
Methylparaben	46.3 (38.4, 55.8)	31.7 (26.4, 38.1)^b^	20.8 (16.5, 26.1)^b^	0.1 (0.1, 0.2)
Propylparaben	3.9 (3, 5)	2.3 (1.8, 2.8)^b^	1.7 (1.3, 2.3)^b^	0.2 (0.1, 0.3)
∑Parabens^c^	0.4 (0.3, 0.5)	0.3 (0.2, 0.3)^b^	0.2 (0.1, 0.2)^b^	0.1 (0.1, 0.2)
Phthalates				
MCP	42.5 (38.8, 46.5)	29.6 (27, 32.4)^b^	32.6 (29.6, 35.9)^b^	0.1 (0.01, 0.19)
MCPP	3.3 (3, 3.7)	2.8 (2.5, 3.2)^b^	2.7 (2.4, 3.1)^b^	0.1 (0.05, 0.23)
MECPP	86.9 (79, 95.5)	46.3 (42.5, 50.4)^b^	36.8 (33.2, 40.7)^b^	0.1 (0, 0.15)
MEHHP	43.2 (39, 47.7)	22.8 (21.0, 24.8)^b^	19.2 (17.3, 21.3)^b^	0.1 (0, 0.17)
MEHP	4.4 (4, 4.9)	3.2 (2.9, 3.5)^b^	2.9 (2.6, 3.2)^b^	0.3 (0.21, 0.39)
MEOHP	26.1 (23.7, 28.9)	14.5 (13.3, 15.9)^b^	12.2 (11.0, 13.5)^b^	0.1 (0.02, 0.2)
MEP	168.3 (147, 192.7)	108.2 (95.0, 123.1)^b^	95.4 (81.9, 111.1)^b^	0.1 (0, 0.18)
MICP	39.5 (35.9, 43.3)	34 (31.3, 37)^b^	28 (25.7, 30.5)^b^	0.3 (0.16, 0.35)
∑DEHP^c^	0.5 (0.5, 0.6)	0.3 (0.3, 0.3)^b^	0.2 (0.2, 0.3)^b^	0.1 (0, 0.16)
∑High-MWP^c^	0.6 (0.5, 0.7)	0.3 (0.3, 0.4)^b^	0.3 (0.2, 0.3)^b^	0.1 (0.02, 0.2)
∑Low-MWP^c^	1.5 (1.3, 1.6)	1 (0.9, 1.1)^b^	0.9 (0.8, 1)^b^	0.1 (0, 0.18)

a Tanner stages correspond to pubertal breast development stages B1 (pre-pubertal) and B4 (late puberty)

b Significant difference (p < 0.05) in urinary EDC biomarker concentration compared to Tanner Stage B1 (reference) based on linear mixed models

c Concentrations are presented in μg/g creatinine except for ∑Parabens, ∑Phenols, ∑DEHP, ∑High-MWP, ∑Low-MWP (μmol/g creatinine).

**Table 3:** Relative change in log_10_ percent FGV measured at 2-years post-menarche (95 % CI) associated with log_10_ (ng/ml) increase in urinary phenol, paraben, and phthalate biomarkers measured at Tanner Breast Stage 1 (B1), Tanner Breast Stage 4, and 1-year post-menarche^a^.

	EDC Biomarker Study Time Point			
	Tanner Stage B1^b^	Tanner Stage B4^b^	1-Year Post-Menarche	Interaction (p-value)^c^
Phenols				
Benzophenone-3	−0.024 (−0.048, 0.00)	0.004 (−0.012, 0.021)	0.006 (−0.015, 0.026)	0.15
BPA	0.005 (−0.029, 0.039)	0.001 (−0.026, 0.028)	−0.02 (−0.063, 0.023)	0.784
BPS	−0.016 (−0.055, 0.024)	0.019 (−0.007, 0.045)	0.011 (−0.019, 0.04)	0.395
Triclosan	0.009 (−0.009, 0.026)	0.009 (−0.007, 0.025)	−**0.025 (−0.041, −0.008)**	**0.007** ^d^
∑Phenols	−0.016 (−0.031, 0.00)	−0.001 (−0.014, 0.013)	0.006 (−0.01, 0.022)	0.17
Parabens				
Methylparaben	−0.017 (−0.033, −0.002)	0 (−0.014, 0.014)	0.006 (−0.01, 0.022)	0.116
Ethylparaben	0.002 (−0.012, 0.017)	−0.006 (−0.021, 0.01)	0.007 (−0.011, 0.024)	0.432
Propylparaben	−0.009 (−0.021, 0.003)	−0.001 (−0.012, 0.01)	−0.002 (−0.016, 0.011)	0.677
∑Parabens^c^	−0.005 (−0.028, 0.017)	0.007 (−0.012, 0.025)	−0.008 (−0.029, 0.014)	0.76
Phthalates				
MBP	−0.029 (−0.061, 0.003)	0.017 (−0.01, 0.044)	−0.022 (−0.067, 0.024)	0.419
MCPP	−0.004 (−0.034, 0.027)	0.006 (−0.015, 0.027)	0.004 (−0.023, 0.03)	0.978
MECPP	0.016 (−0.015, 0.046)	0.013 (−0.016, 0.042)	0.007 (−0.032, 0.047)	0.910
MEHHP	0.01 (−0.018, 0.037)	0.017 (−0.013, 0.046)	0.006 (−0.033, 0.045)	0.996
MEHP	0.009 (−0.018, 0.036)	0.013 (−0.012, 0.039)	−0.01 (−0.045, 0.024)	0.705
MEOHP	0.01 (−0.017, 0.038)	0.016 (−0.013, 0.045)	0.01 (−0.029, 0.049)	0.985
MEP	0.013 (−0.007, 0.033)	−0.002 (−0.021, 0.017)	−0.001 (−0.024, 0.023)	0.422
MiBP	−0.02 (−0.052, 0.012)	0.01 (−0.022, 0.042)	0.002 (−0.045, 0.048)	0.742
∑DEHP	0.013 (−0.016, 0.043)	0.015 (−0.015, 0.044)	0.007 (−0.033, 0.047)	0.959
∑High-MWP	0.011 (−0.019, 0.04)	0.01 (−0.02, 0.041)	0.007 (−0.032, 0.045)	0.941
∑Low-MWP	0.014 (−0.012, 0.04)	0.001 (−0.023, 0.024)	−0.002 (−0.033, 0.029)	0.599

a Estimating average change (β) in %FGV associated with EDC biomarkers across study time points using a multivariable generalized estimation equations (GEE) model. Adjusted models include creatinine, maternal education, age at study time point, body fat percentage at study time point, and average daily caloric intake at study time point. Associations in which the 95 %CI does not include 0 are in bold.

b Tanner stages correspond to pubertal breast development stages B1 (pre-pubertal) and B4 (late puberty)

c P-value for interaction between EDC biomarker concentration and study time point on %FGV (F test); p < 0.05 are in bold.

d False Discovery Rate (FDR) < 0.05 from Benjamini-Hochberg adjusted p-values

**Table 4:** Relative change in log_10_ absolute FGV measured at 2-years post-menarche (95 % CI) associated with log_10_ (ng/ml) increase in urinary phenol, paraben, and phthalate biomarkers measured at Tanner Breast Stage 1 (B1), Tanner Breast Stage 4, and 1-year post-menarche^a^.

	EDC Biomarker Study Time Point			
	Tanner Stage B1^b^	Tanner Stage B4^b^	1-Year Post-Menarche	Interaction (p-value)^c^
Phenols				
Benzophenone-3	−0.024 (−0.074, 0.025)	−0.009 (−0.037, 0.019)	0.004 (−0.036, 0.043)	0.311
BPA	−0.018 (−0.078, 0.042)	0.006 (−0.04, 0.053)	−0.004 (−0.083, 0.076)	0.194
BPS	−0.007 (−0.073, 0.058)	0.004 (−0.04, 0.047)	0.025 (−0.035, 0.086)	0.654
Triclosan	0.001 (−0.032, 0.034)	−0.013 (−0.043, 0.016)	−0.02 (−0.052, 0.011)	0.969
∑Phenols	−0.013 (−0.041, 0.014)	−0.013 (−0.035, 0.009)	0.018 (−0.012, 0.048)	0.141
Parabens				
Methylparaben	−0.015 (−0.044, 0.013)	−0.012 (−0.034, 0.009)	0.016 (−0.014, 0.046)	0.167
Ethylparaben	0.008 (−0.021, 0.036)	−0.012 (−0.037, 0.013)	0.021 (−0.01, 0.052)	0.231
Propylparaben	−0.013 (−0.033, 0.008)	−0.012 (−0.03, 0.006)	0.02 (−0.005, 0.046)	0.068
∑Parabens^c^	−0.011 (−0.057, 0.035)	−0.016 (−0.046, 0.015)	−0.002 (−0.041, 0.037)	0.517
Phthalates				
MBP	−**0.087 (−0.15, −0.024)**	0.028 (−0.016, 0.071)	0.052 (−0.028, 0.133)	**0.001** ^d^
MCPP	0.00 (−0.055, 0.056)	0.024 (−0.018, 0.067)	0.02 (−0.028, 0.068)	0.249
MECPP	0.014 (−0.043, 0.071)	**0.055 (0.006, 0.104)**	0.052 (−0.022, 0.126)	0.104
MEHHP	0.001 (−0.05, 0.051)	0.05 (−0.001, 0.1)	0.053 (−0.021, 0.126)	**0.041**
MEHP	0.007 (−0.043, 0.058)	0.039 (−0.005, 0.082)	0.04 (−0.023, 0.102)	0.056
MEOHP	0.004 (−0.047, 0.055)	0.047 (−0.003, 0.097)	0.053 (−0.019, 0.125)	0.051
MEP	0.014 (−0.022, 0.051)	0.012 (−0.02, 0.045)	0.011 (−0.031, 0.054)	0.701
MiBP	−0.054 (−0.113, 0.006)	0.001 (−0.049, 0.052)	0.04 (−0.045, 0.124)	**0.024**
∑DEHP	0.009 (−0.046, 0.064)	**0.053 (0.003, 0.104)**	0.054 (−0.021, 0.129)	0.068
∑High-MWP	0.006 (−0.05, 0.062)	0.042 (−0.014, 0.099)	0.05 (−0.022, 0.123)	0.094
∑Low-MWP	−0.006 (−0.052, 0.041)	0.019 (−0.021, 0.058)	0.029 (−0.026, 0.084)	0.136

a Estimating average change (β) in aFGV associated with EDC biomarkers across study time points using a multivariable generalized estimation equations (GEE) model. Adjusted models include creatinine, maternal education, age at study time point, body fat percentage at study time point, and average daily caloric intake at study time point. Associations in which the 95 %CI does not include 0) are in bold.

b Tanner stages correspond to pubertal breast development stages B1 (pre-pubertal) and B4 (late puberty)

c P-value for interaction between EDC biomarker concentration and study time point on %FGV (F test); p < 0.05 are in bold.

d False Discovery Rate (FDR) < 0.05 from Benjamini-Hochberg adjusted p-values

**Table 5:** Relative change in log_10_ total breast volume measured at 2-years post-menarche (95 % CI) associated with log_10_ (ng/ml) increase in urinary phenol, paraben, and phthalate biomarkers measured at Tanner Breast Stage 1 (B1), Tanner Breast Stage 4, and 1-year post-menarche^a^.

	EDC Biomarker Study Time Point			
	Tanner Stage B1^b^	Tanner Stage B4^b^	1-Year Post-Menarche	Interaction (p-value)^c^
Phenols				
Benzophenone-3	0 (−0.039, 0.04)	−0.013 (−0.04, 0.013)	−0.002 (−0.036, 0.031)	0.725
BPA	−0.029 (−0.082, 0.023)	0.005 (−0.037, 0.046)	0.014 (−0.036, 0.065)	**0.019**
BPS	0.005 (−0.045, 0.055)	−0.014 (−0.06, 0.033)	0.015 (−0.036, 0.066)	0.724
Triclosan	−0.008 (−0.036, 0.02)	−0.022 (−0.046, 0.002)	0.004 (−0.021, 0.028)	0.27
∑Phenols	0.003 (−0.022, 0.028)	−0.012 (−0.03, 0.006)	0.011 (−0.015, 0.036)	0.362
Parabens				
MEPB	0.003 (−0.023, 0.028)	−0.012 (−0.03, 0.006)	0.008 (−0.017, 0.034)	0.444
ETPB	0.007 (−0.017, 0.031)	−0.007 (−0.029, 0.015)	0.014 (−0.012, 0.04)	0.45
PRPB	−0.003 (−0.022, 0.015)	−0.011 (−0.026, 0.004)	**0.021 (0.001, 0.041)**	**0.038**
∑Parabens	−0.006 (−0.042, 0.03)	−0.022 (−0.049, 0.005)	0.005 (−0.026, 0.037)	0.255
Phthalates				
MBP	−**0.058 (−0.108, −0.007)**	0.01 (−0.03, 0.05)	0.065 (−0.017, 0.147)	**0.003**
MCPP	0.007 (−0.043, 0.056)	0.02 (−0.012, 0.052)	0.014 (−0.027, 0.054)	0.207
MECPP	0.001 (−0.048, 0.051)	**0.045 (0.001, 0.089)**	0.043 (−0.009, 0.095)	**0.03**
MEHHP	−0.007 (−0.048, 0.035)	0.036 (−0.009, 0.082)	0.045 (−0.005, 0.096)	**0.012**
MEHP	−0.002 (−0.043, 0.039)	0.022 (−0.015, 0.059)	**0.049 (0.001. 0.098)**	**0.017**
MEOHP	−0.004 (−0.046, 0.038)	0.034 (−0.01, 0.079)	0.042 (−0.009, 0.093)	**0.017**
MEP	0.00 (−0.032, 0.033)	0.014 (−0.012, 0.041)	0.012 (−0.02, 0.045)	0.244
MiBP	−0.034 (−0.079, 0.011)	−0.008 (−0.05, 0.033)	0.025 (−0.041, 0.092)	0.055
∑DEHP	−0.002 (−0.049, 0.045)	0.042 (−0.004, 0.087)	0.046 (−0.007, 0.098)	**0.019**
∑High-MWP	−0.002 (−0.05, 0.045)	0.035 (−0.011, 0.081)	0.042 (−0.009, 0.093)	**0.032**
∑Low-MWP	−0.021 (−0.062, 0.02)	0.018 (−0.013, 0.05)	0.028 (−0.014, 0.07)	**0.021**

a Estimating average change (β) in tBV associated with EDC biomarkers across study time points using a multivariable generalized estimation equations (GEE) model. Adjusted models include creatinine, maternal education, age at study time point, body fat percentage at study time point, and average daily caloric intake at study time point. Associations in which the 95 % CI does not include 0 are in bold.

b Tanner stages correspond to pubertal breast development stages B1 (pre-pubertal) and B4 (late puberty)

c P-value for interaction between EDC biomarker concentration and study time point on %FGV (F test); p < 0.05 are in bold.

d False discovery rate (FDR) < 0.05 from Benjamini-Hochberg adjusted p-values

## Data Availability

Data will be made available on request.

## Abbreviations:

EDC: Endocrine disrupting chemicals
BPA: Bisphenol A
MEP: Monoethyl phthalate
MEHP: Mono(2-ethylhexyl) phthalate
DEHP: di(2-ethylhexyl) phthalate
BMI: Body mass index
B1: Tanner breast stage 1
B4: Tanner breast stage 4
MCOP: Monocarboxy-isooctyl phthalate
GOCS: Growth and Obesity Cohort Study
INTA: Instituto de Nutrición y Tecnología de los Alimentos
BPF: Bisphenol F
BPS: Bisphenol S
MECPP: Mono(2-ethyl-5-carboxypentyl) phthalate
MEHHP: Mono(2-ethyl-5-hydroxyhexyl) phthalate
MEOHP: Mono(2-ethyl-5-oxohexyl) phthalate
MiBP: Mono-isobutyl phthalate
MBP: Mono-*n*-butyl phthalate
MCPP: Mono-3-carboxypropyl phthalate
LOD: Limit of detection
MWP: Molecular weight phthalate
DXA: Dual energy X-ray absorptiometry
FGV: Fibroglandular volume
BV: Breast volume
ICC: Intraclass correlation coefficient
LMM: Linear mixed models
GEE: Generalized estimating equation
FDR: False discovery rate
UPF: Ultra-processed foods
