# Supplementary material for: Variability in urinary phthalates, phenols, and parabens across childhood and relation to adolescent breast composition in Chilean girls
Source: Environ Int. Author manuscript; Available in PMC 2023 Sep 23. (PMC10517447; doi:10.1016/j.envint.2022.107586)
Supplement: Appendix A. Supplemental Material [file NIHMS1930548-supplement-Appendix_A__Supplemental_Material.docx]

**Supplemental Figures and Tables**

Supplemental Table 1. Comparison of characteristics for girls in the Growth and Obesity Cohort Study with breast assessments (n=366) and without breast assessments (n=159)

| **Characteristic** | **Included in the analysis^a^ (n=366)** | | | **Excluded from the analysis (n=159)** | | |
| --- | --- | --- | --- | --- | --- | --- |
|  | **Study Time Point^b^** | | | **Study Time Point** | | |
|  | Tanner Stage B1 | Tanner Stage B4 | 1-Year  Post-Menarche | Tanner Stage B1 | Tanner Stage B4 | 1-Year  Post-Menarche |
|  | (n=293) | (n=333) | (n=232) | (n=122) | (n=115) | (n=34) |
| Urine sample provided |  |  |  |  |  |  |
| B1 | 293 (100.0) | 261 (78.4) | 197 (84.9) | 122 (100.0) | 79 (68.7) | 28 (82.4) |
| B4 | 261 (89.1) | 333 (100.0) | 219 (94.4) | 79 (64.8) | 115 (100.0) | 28 (82.4) |
| 1Y PM | 197 (67.2) | 219 (65.8) | 232 (100.0) | 28 (23.0) | 28 (24.3) | 34 (100.0) |
| Age, years (mean (SD)) | 7.87 (0.45) | 11.38 (0.88) | 13.40 (0.82) | 7.92 (0.50) | 11.24 (0.98) | 14.01 (1.18) |
| Age at menarche, years (mean (SD)) | 12.09 (0.91) | 12.04 (0.89) | 12.42 (0.76) | 12.14 (1.08) | 11.86 (1.09) | 12.88 (1.09) |
| BMI Z-score (mean (SD)) | 0.85 (1.10) | 0.88 (1.10) | 0.86 (1.11) | 0.86 (1.17) | 0.96 (1.07) | 0.67 (1.08) |
| Body fat percentage (mean (SD)) | 25.58 (4.41) | 26.93 (5.11) | 30.62 (5.61) | 25.91 (5.02) | 26.64 (5.40) | 29.75 (5.85) |
| Body fat percentage (count (%)) |  |  |  |  |  |  |
| Underfat/Normal | 155 (54.4) | 198 (62.3) | 55 (40.1) | 69 (57.5) | 74 (67.3) | 11 (47.8) |
| Overfat | 82 (28.8) | 68 (21.4) | 39 (28.5) | 28 (23.3) | 20 (18.2) | 2 (8.7) |
| Obese | 48 (16.8) | 52 (16.4) | 43 (31.4) | 23 (19.2) | 16 (14.5) | 10 (43.5) |
| Maternal education (%) |  |  |  |  |  |  |
| Secondary education or less | 236 (80.5) | 271 (81.4) | 192 (82.8) | 96 (78.7) | 85 (73.9) | 22 (64.7) |
| Greater than secondary education | 57 (19.5) | 62 (18.6) | 40 (17.2) | 26 (21.3) | 30 (26.1) | 12 (35.3) |
| Duration of breast feeding (count (%)) |  |  |  |  |  |  |
| < 3 months | 90 (30.7) | 107 (32.1) | 74 (31.9) | 35 (28.7) | 31 (27.0) | 13 (38.2) |
| 3-6 months | 171 (58.4) | 188 (56.5) | 134 (57.8) | 77 (63.1) | 69 (60.0) | 17 (50.0) |
| > 6 months | 32 (10.9) | 38 (11.4) | 24 (10.3) | 10 (8.2) | 15 (13.0) | 4 (11.8) |
| Birth mode (%) |  |  |  |  |  |  |
| Caesarean | 85 (29.0) | 91 (27.3) | 73 (31.5) | 50 (41.0) | 32 (27.8) | 7 (20.6) |
| Vaginal | 208 (71.0) | 242 (72.7) | 159 (68.5) | 72 (59.0) | 83 (72.2) | 27 (79.4) |
| Average caloric intake, kCal (mean (SD)) | 1878.81 (490.73) | 1873.00 (457.64) | 1745.07 (446.37) | 1906.21 (544.87) | 1871.09 (465.24) | 1759.18 (530.93) |

a Only girls with breast assessments at 2-years post-menarche were included in the analysis

b Tanner stages correspond to pubertal breast development stages B1 (pre-pubertal) and B4 (late puberty)


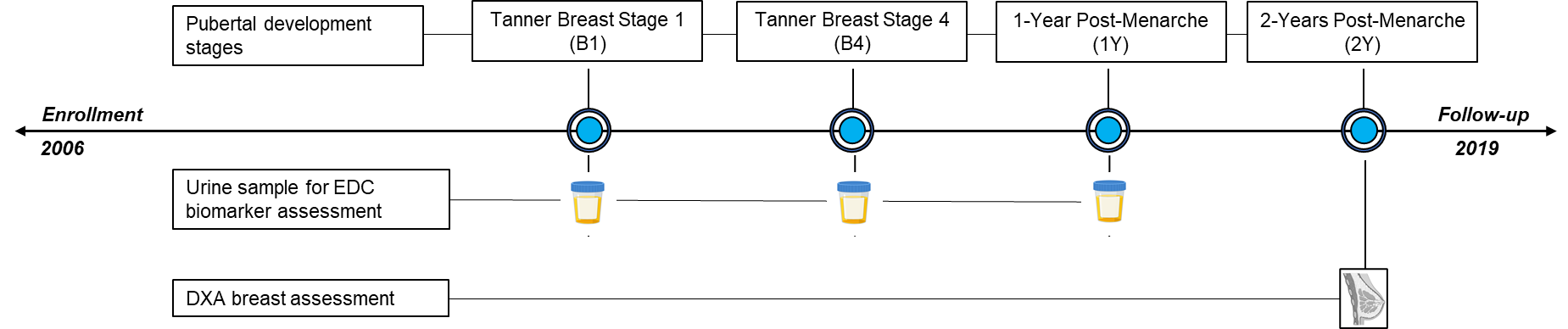


Supplemental Figure 1. Exposure and outcome assessment time points during follow-up in the Growth and Obesity Cohort Study.


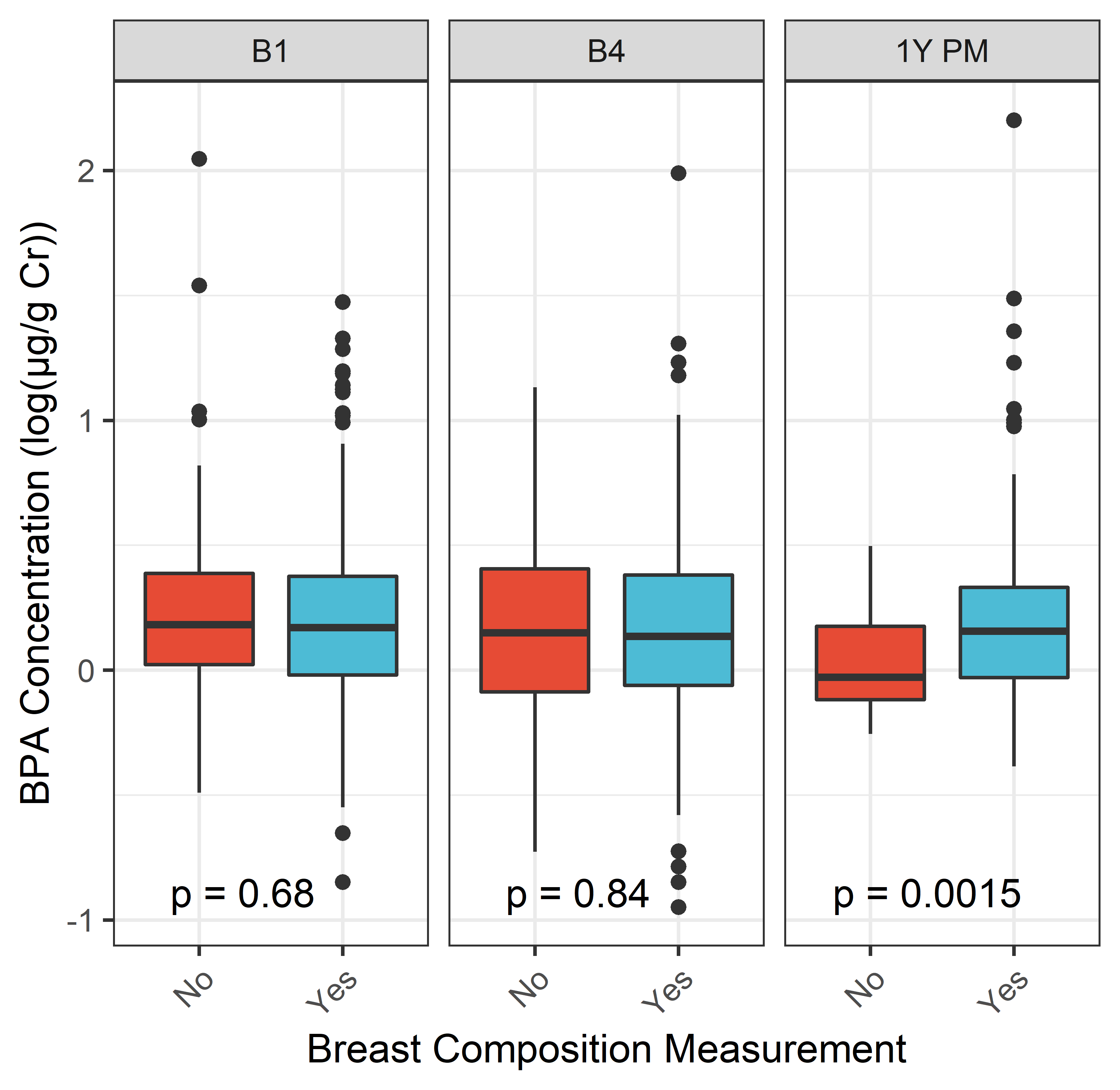


Supplemental Figure 2. Distribution of BPA concentration (log_10_-transformed and creatinine-adjusted) at Tanner Breast Stage B1 (pre-pubertal), B4 (late puberty), or 1-year post-menarche for girls with breast composition assessments and those without breast composition assessments. Only girls with breast composition assessments were included in the analysis. P-values were produced from non-parametric t-test for two-sample comparison of means.


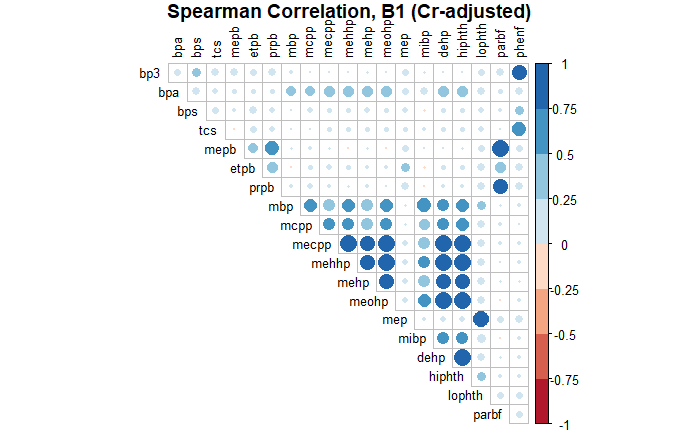


Supplemental Figure 3. Plot of Spearman correlation for EDC biomarker concentrations (creatinine-adjusted) at Tanner Breast Stage B1 (pre-puberty) among 293 girls participating in the Growth and Obesity Cohort Study in Santiago, Chile. Acronyms: Bbenzophenone-3 (bp3), bisphenol-A (bpa), bisphenol S (bps), triclosan (tcs), ethylparaben (etpb), methylparaben (mepb), propylparaben (prpb), mono(2-ethyl-5-carboxypentyl phthalate (mecpp), mono(2-ethyl-5-hydroxyhexyl) phthalate (mehhp), mono(2-ethylhexyl) phthalate (mehp), mono(2-ethyl-5-oxohexyl) phthalate (meohp), monoethyl phthalate (mep), mono-isobutyl phthalate (mibp), mono-n-butyl phthalate (mbp), mono-3-carboxypropyl phthalate (mcpp), oxidative di(2-ethylhexyl) phthalates (dehp), high-molecular weight phthalates (hiphth), low-molecular weight phthalates (lophth), parabens (parbf), phenols (phenf).


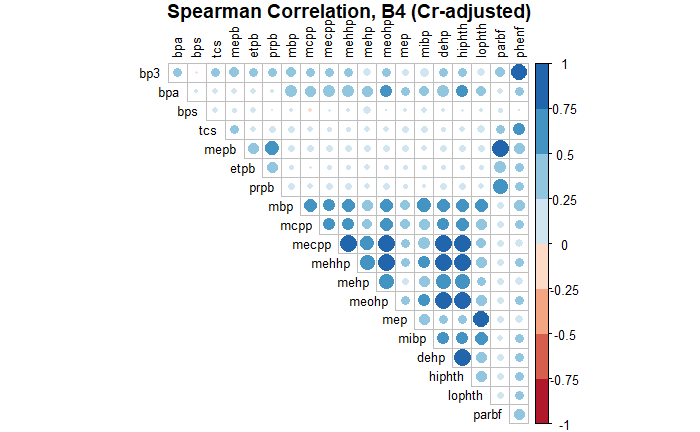


Supplemental Figure 4. Plot of Spearman correlation for EDC biomarker concentrations (creatinine-adjusted) at Tanner Breast Stage B4 (late puberty) among 333 girls participating in the Growth and Obesity Cohort Study in Santiago, Chile. Acronyms: Bbenzophenone-3 (bp3), bisphenol-A (bpa), bisphenol S (bps), triclosan (tcs), ethylparaben (etpb), methylparaben (mepb), propylparaben (prpb), mono(2-ethyl-5-carboxypentyl phthalate (mecpp), mono(2-ethyl-5-hydroxyhexyl) phthalate (mehhp), mono(2-ethylhexyl) phthalate (mehp), mono(2-ethyl-5-oxohexyl) phthalate (meohp), monoethyl phthalate (mep), mono-isobutyl phthalate (mibp), mono-n-butyl phthalate (mbp), mono-3-carboxypropyl phthalate (mcpp), oxidative di(2-ethylhexyl) phthalates (dehp), high-molecular weight phthalates (hiphth), low-molecular weight phthalates (lophth), parabens (parbf), phenols (phenf)


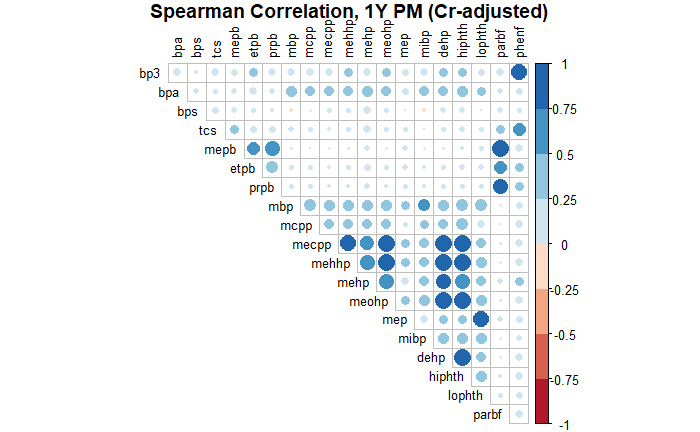


Supplemental Figure 5. Plot of Spearman correlation for EDC biomarker concentrations (creatinine-adjusted) at 1-year post-menarche among 232 girls participating in the Growth and Obesity Cohort Study in Santiago, Chile. Acronyms: Bbenzophenone-3 (bp3), bisphenol-A (bpa), bisphenol S (bps), triclosan (tcs), ethylparaben (etpb), methylparaben (mepb), propylparaben (prpb), mono(2-ethyl-5-carboxypentyl phthalate (mecpp), mono(2-ethyl-5-hydroxyhexyl) phthalate (mehhp), mono(2-ethylhexyl) phthalate (mehp), mono(2-ethyl-5-oxohexyl) phthalate (meohp), monoethyl phthalate (mep), mono-isobutyl phthalate (mibp), mono-n-butyl phthalate (mbp), mono-3-carboxypropyl phthalate (mcpp), oxidative di(2-ethylhexyl) phthalates (dehp), high-molecular weight phthalates (hiphth), low-molecular weight phthalates (lophth), parabens (parbf), phenols (phenf)

| 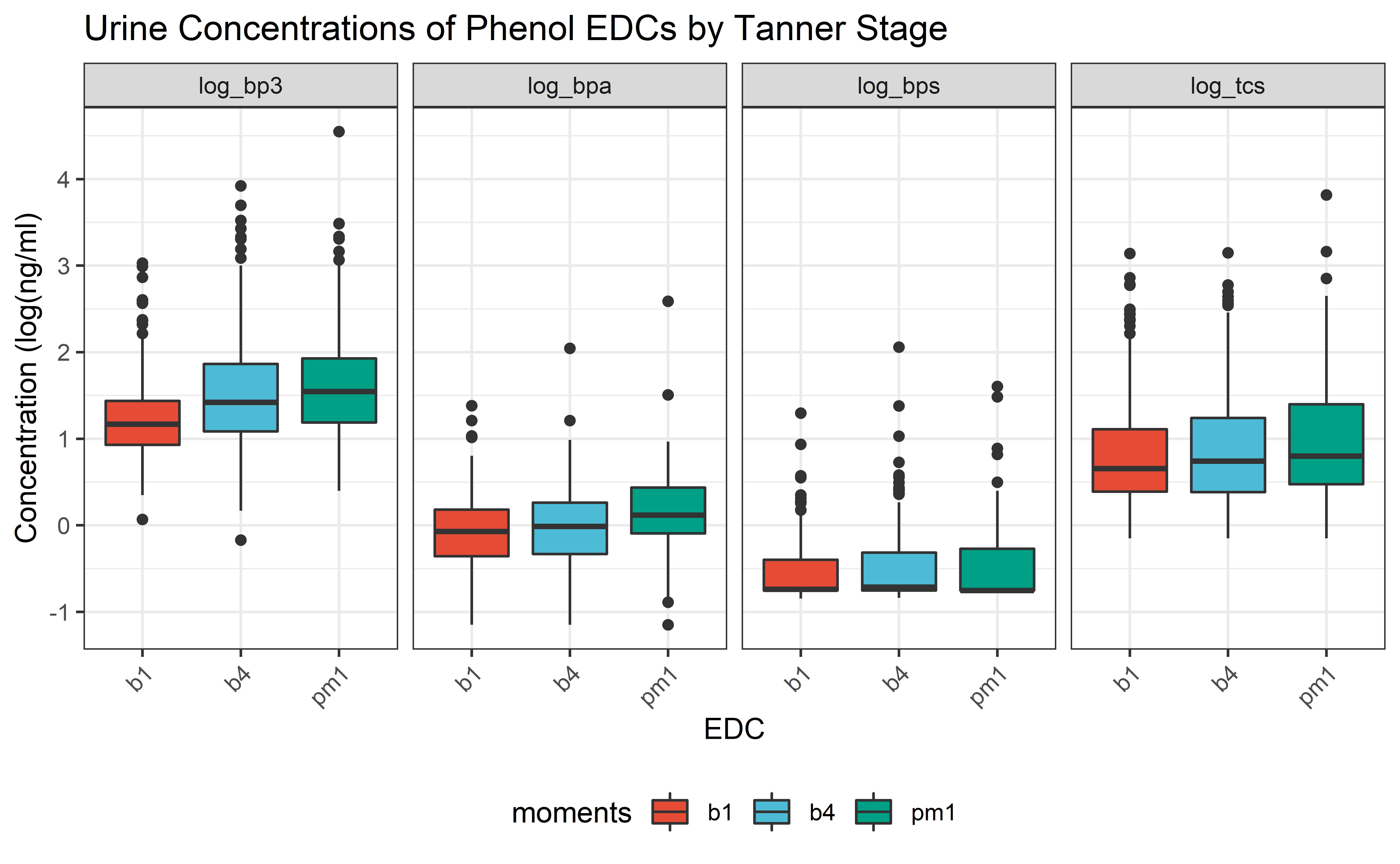 | 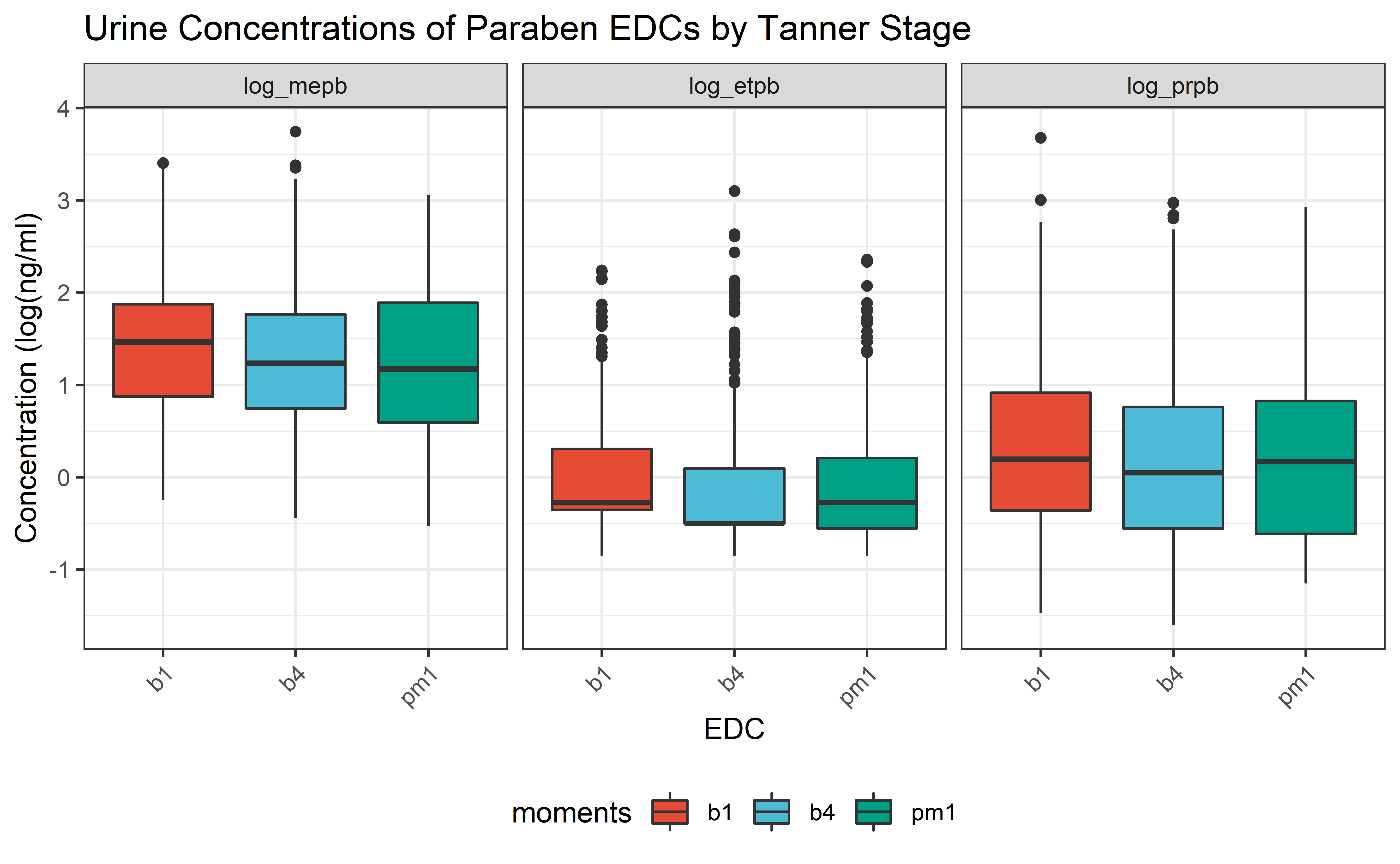 |
| --- | --- |
| 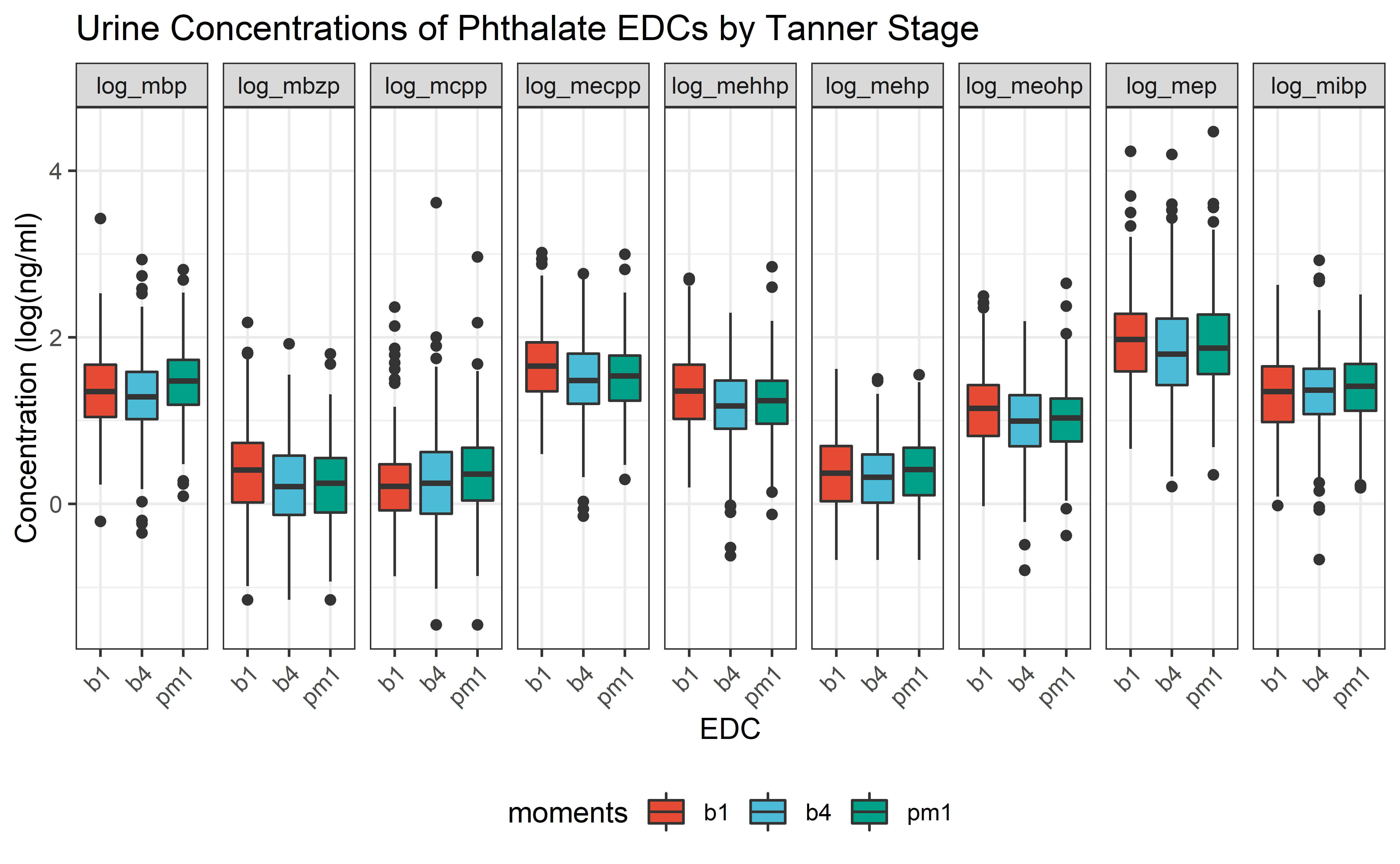 | 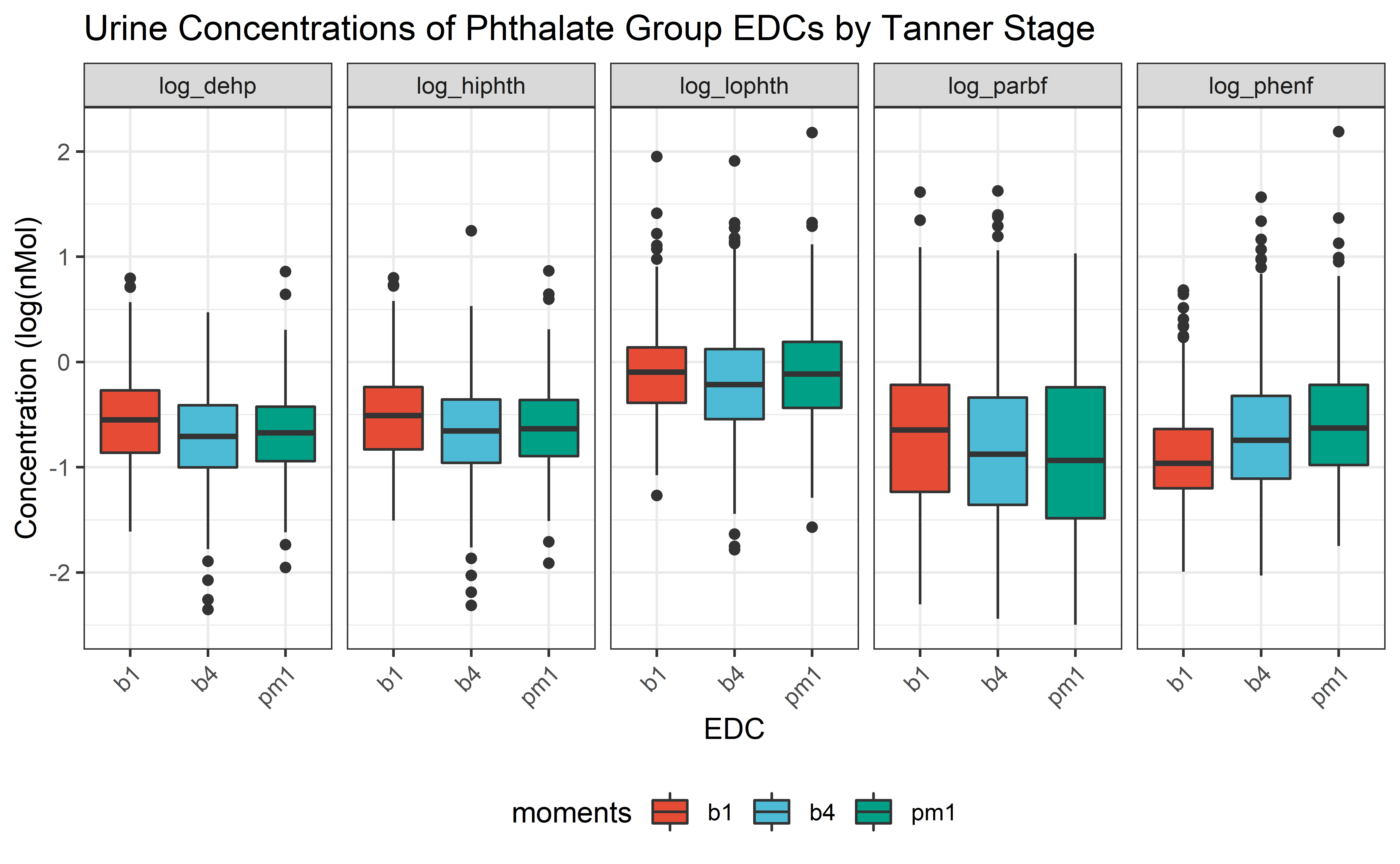 |

Supplemental Figure 6. Box plots of log-transformed urine concentrations of endocrine disrupting chemicals (EDCs; log(ng/ml)) by Tanner Stage (‘moments’) among 366 girls participating in the Growth and Obesity Cohort Study in Santiago, Chile. Acronyms: Bbenzophenone-3 (bp3), bisphenol-A (bpa), bisphenol S (bps), triclosan (tcs), ethylparaben (etpb), methylparaben (mepb), propylparaben (prpb), mono(2-ethyl-5-carboxypentyl phthalate (mecpp), mono(2-ethyl-5-hydroxyhexyl) phthalate (mehhp), mono(2-ethylhexyl) phthalate (mehp), mono(2-ethyl-5-oxohexyl) phthalate (meohp), monoethyl phthalate (mep), mono-isobutyl phthalate (mibp), mono-n-butyl phthalate (mbp), mono-3-carboxypropyl phthalate (mcpp)
